# Supplementary material for: Nanomaterial Exposure, Extracellular Vesicle Biogenesis and Adverse Cellular Outcomes: A Scoping Review
Source: Nanomaterials (Basel). 2022 Apr 6;12(7):1231. doi: 10.3390/nano12071231 (PMC9000848; doi:10.3390/nano12071231)
Supplement: Supplementary file 1 [file nanomaterials-12-01231-s001.zip › nanomaterials-1650688-supplementary.pdf]

nanomaterials MDPI

Supplementary Materials

Nanomaterial Exposure, Extracellular Vesicle Biogenesis and Adverse Cellular Outcomes: A Scoping Review

Thais S. M. Lima [1,2], Wanderson Souza [1,2], Luths R. O. Geaquinto [1,2], Priscila L. Sanches [1,3], Ewa. L. Stepień [4], João Meneses [5], Eli Fernández-de Gortari [5], Nicole Meisner-Kober [6], Martin Himly [6], José M. Granjeiro [1,2,3,7,*] and Ana R. Ribeiro [2,5,*]

[1] Directory of Life Sciences Applied Metrology, National Institute of Metrology Quality and Technology, 25250-020,Rio de Janeiro, Brazil; thaismellolima@gmail.com (T.S.M.L.); wstudzel@gmail.com (W.S.); luthsgeaquinto@gmail.com (L.R.O.G.); plaviolasanches@gmail.com (P.L.S.)
[2] Postgraduate Program in Biotechnology, National Institute of Metrology Quality and Technology, 25250-020, Rio de Janeiro, Brazil
[3] Postgraduate Program in Translational Biomedicine, University Grande Rio, 25071-202Duque de Caxias, Brazil
[4] Faculty of Physics, Astronomy, and Applied Computer Science, Jagiellonian University, 30-348 Kraków, Poland; e.stepien@uj.edu.pl (E.L.S)
[5] NanoSafety Group, International Iberian Nanotechnology Laboratory, 4715-330 Braga, Portugal; joao.meneses@inl.int (J.M.); eli.fernandez@inl.int (E.F.-d.G.)
[6] Department of Biosciences & Medical Biology, University of Salzburg, 5020, Salzburg ,Austria; nicole.meisner-kober@plus.ac.at (N.M.-K.); martin.himly@plus.ac.at (M.H.)
[7] Dental School, Fluminense Federal University, 24020-140 Niterói, Brazil
* Correspondence: jmgranjeiro@gmail.com (J.M.G.); ana.ribeiro@inl.int (A.R.R.)

Table S1. Search strategy used in the gray literature databases included in the study.

Database	Keywords
Grey Literature Report	(“exosome” OR “EVs” OR “Extracellular vesicles” OR “Apoptotic bodies” OR "MVBs" OR “Multivesicular bodies”) AND (“nanoparticles” OR “NPs” OR “nanomaterials”) AND (“toxicology” OR “toxicity” OR “inflammation” OR “composition” OR “biological function” OR “biogenesis” OR “cellular biology” OR “cargo” OR “effects” OR “signaling” OR “communication” OR “cellular response” OR “biological activities”)
World Health Organization (Library Catalog)	(“exosome” OR “EVs” OR “Extracellular vesicles” OR “Apoptotic bodies” OR "MVBs" OR “Multivesicular bodies”) AND (“nanoparticles” OR “NPs” OR

	“nanomaterials”) AND (“toxicology” OR “toxicity” OR “inflammation” OR “composition” OR “biological function” OR “biogenesis” OR “cellular biology” OR “cargo” OR “effects” OR “signaling” OR “communication” OR “cellular response” OR “biological activities”)
Networked Digital Library of Theses and Dissertations	(“exosome” OR “EVs” OR “Extracellular vesicles” OR “Apoptotic bodies” OR "MVBs" OR “Multivesicular bodies”) AND (“nanoparticles” OR “NPs” OR “nanomaterials”) AND (“toxicology” OR “toxicity” OR “inflammation” OR “composition” OR “biological function” OR “biogenesis” OR “cellular biology” OR “cargo” OR “effects” OR “signaling” OR “communication” OR “cellular response” OR “biological activities”)
CADTH Evidence Driven	(“exosome” OR “EVs” OR “Extracellular vesicles” OR “Apoptotic bodies” OR "MVBs" OR “Multivesicular bodies”) AND (“nanoparticles” OR “NPs” OR “nanomaterials”) AND (“toxicology” OR “toxicity” OR “inflammation” OR “composition” OR “biological function” OR “biogenesis” OR “cellular biology” OR “cargo” OR “effects” OR “signaling” OR “communication” OR “cellular response” OR “biological activities”)

Table S2. Inclusion and exclusion criteria.

PECO	Inclusion	Exclusion
Population	In vitro, in vivo, ex vivo studies and clinical trials that isolated extracellular vesicles after exposure to any type of nanomaterial.	Extracellular vesicles isolated from experimental models that are not similar to humans such as: plants, viruses, fungi, bacteria and aquatic animals for isolation of extracellular vesicles.
Exposure	Exhibition of any type of nanomaterial.	Exposure to materials on the micrometric scale; Exposure to drugs that use nanomaterials only as a vehicle and that do not present any type of response derived only from exposure to the nanomaterial.
Comparison	Studies that isolated extracellular vesicles without exposure to any type of nanomaterial (control).	Studies that did not analyze extracellular vesicles without exposure to any type of nanomaterial.

Outcome	Cellular responses mediated by extracellular vesicles induced after exposure to nanomaterials;<br><br>Changes in extracellular vesicles induced by exposure to any type of nanomaterial such as number, morphology, content, size etc.	Studies focusing on extracellular vesicle isolation method;<br>Studies that do not show cellular responses mediated by extracellular vesicles<br>Studies focusing on improving techniques;<br>Studies that demonstrate the release of apoptotic bodies during cell death, but do not show cellular responses derived from cell communication mediated by this extracellular vesicle or changes such as number, morphology, content, size, etc.
Type of publication	Only articles with original data and systematic or scope-based review articles based on original articles.	Articles without original data such as editorials, narrative reviews, book chapters, letters and comments.

Table S3. Initial information regarding the included studies.

Reference	Title	DOI	Year	Country	Periodical
[20]	Cellular communication via nanoparticle-transporting biovesicles	10.2217/nnm.13.57	2014	United States	Nanomedicine
[22]	Nanoparticle-Induced Exosomes Target Antigen-Presenting Cells to Initiate Th1-Type Immune Activation	10.1002/smll.201200381	2012	China	Small
[24]	Exosomal miRNA Profiling to Identify Nanoparticle Phagocytic Mechanisms	10.1002/smll.201704008	2018	China	Small

[25]	Human primary macrophages scavenge AuNPs and eliminate it through exosomes. A natural shuttling for nanomaterials	10.1016/j.ejpb.2019.02.014	2019	Italy	European Journal of Pharmaceutics and Biopharmaceutics
[26]	The Fate of SWCNTs in Mouse Peritoneal Macrophages: Exocytosis, Biodegradation, and Sustainable	10.3389/fbioe.2020.00211	2020	China	Frontiers in Bioengineering and Biotechnology
[39]	Effects of subtoxic concentrations of TiO_2 and ZnO nanoparticles on human lymphocytes, dendritic cells and exosome production	10.1016/j.taap.2012.07.021	2012	Sweden	Toxicology and Applied Pharmacology
[40]	Exosomes Derived from Bone Mesenchymal Stem Cells with the Stimulation of Fe_3O_4 Nanoparticles and Static Magnetic Field Enhance Wound Healing Through Upregulated miR-21-5p	10.2147/IJN.S275650	2020	China	International Journal of Nanomedicine.
[41]	Exosomes as Extrapulmonary Signaling Conveyors for Nanoparticle-Induced Systemic Immune Activation	10.1002/smll.201101708	2012	China	Small

[42]	Calcium phosphate particles stimulate exosome secretion from phagocytes for the enhancement of drug delivery	10.1016/j.colsurfb.2018.07.037	2018	China	Colloids and Surfaces B-Biointerfaces
[43]	Gold nanoparticles change small extracellular vesicle attributes of mouse embryonic stem cells	10.1039/D0NR03598J	2020	China	Nanoscale
[44]	Platinum Nanoparticles Enhance Exosome Release in Human Lung Epithelial Adenocarcinoma Cancer Cells (A549): Oxidative Stress and the Ceramide Pathway are Key Players	10.2147 / IJN.S291138	2021	Korea	Int J Nanomedicine
[45]	Palladium Nanoparticle-Induced Oxidative Stress, Endoplasmic Reticulum Stress, Apoptosis, and Immunomodulation Enhance the Biogenesis and Release of Exosome in Human Leukemia Monocytic Cells (THP-1)	10.2147/IJN.S305269	2021	Korea	Int J Nanomedicine
[46]	Biogenesis of Extracellular Vesicles Produced from Human-Stem-Cell-Derived Cortical Spheroids Exposed to Iron Oxides	10.1021/acsbiomaterials.0c01286	2021	United States	ACS Biomater Sci Eng

[47]	Effect of multi-functional polyhydroxylated polyhedral oligomeric silsesquioxane (POSS) nanoparticles on the angiogenesis and exosome biogenesis in human umbilical vein endothelial cells (HUVECs)	10.1016/j.matdes.2020.109227	2021	Iran	Materials & Design
[48]	Nano-hydroxyapatite accelerates vascular calcification via lysosome impairment and autophagy dysfunction in smooth muscle cells	doi.org/10.1016/j.bioactmat.2021.06.004	2021	China	Bioactive Materials
[49]	Shedding plasma membrane vesicles induced by graphene oxide nanoflakes in brain cultured astrocytes	10.1016/j.carbon.2021.01.142	2021	Italy	Carbon
[50]	Cell membrane disintegration and extracellular vesicle release in a model of different size and charge PAMAM dendrimers effects on cultured endothelial cells	10.1080/17435390.2019.1570373	2019	United States	Nanotoxicoloy

[51]	In situ self-assembly Ag - Fe_3O_4 nanoclusters in exosomes for cancer diagnosis	10.1039/C9TB02610J	2020	China	Journal of Materials Chemistry B

Table S4. Biological models used in the included studies.

Reference	Biological models	Type of assay	Experimental size (n)	Quality control of the biological sample
[20]	Human	In vitro	-	-
[22]	Mouse	In vivo	n = 48	-
[24]	Human	Clinical trial and In vitro	n = 154	-
[25]	Human	In vitro	n = 5	-
[26]	Mouse	In vitro	n = 3	Cell authenticity (CD11b >95%)
[39]	Human	In vitro	n = 6–8	Trypan Blue, (viability > 90%)
[40]	Human	In vitro	n = 3	-
[40]	Human<br>Mouse	In vitro<br>In vivo	Cells (n=3)<br>Mouse (n=24)	-
[42]	Human	In vitro and in vivo	n=6	-

	Mouse			
[43]	Mouse	In vitro	n = 3	
[44]	Human	In vitro	n = 3–8	-
[45]	Human	In vitro	n = 3	-
[46]	Mouse	In vivo	n = 48	-
[47]	Human<br>Mouse	In vitro	n = 6	-
[48]	Camundongo	In vitro	n = 3	-
[49]	Humano	In vitro	n = 3	-
[50]	Humano	In vitro	n = 3	-
[51]	Humano	In vitro	n = 3	-

Table S5. Characteristics of nanomaterials.

Reference	Nanomaterial	Morphology / Crystalline structure	Size	Z Potencial	PDI	Purity	Characterization Methods
[20]	PEI-SPION NPs	-	15 nm	Low density: 4.5 mV<br>High density: 7.7 mV	-	-	-

[22]	MIONs	-	P: 43 nm<br>CM: 43 ± 5 nm	-	-	LPS< 0.25 EU ml^{-1}	TEM, X-ray diffraction, BET
[24]	SiO_2 NPs	-	10–20 nm				-
	Occupational NPs	-	-	-	-	≈ 99,5%	
[25]	AuNPs	-	P: 20 nm<br><br>Water: 20.5 nm (T0h), 20.2 nm (T24h), CM: 19.5 nm (T0h), 19.9 nm (T24h)	-	-	-	ICP-MS, NTA, Laser scanning confocal microscopy (LSCM)
[26]	SWNCTs	Fiber-like	200–1000 nm	−44.1 mV (pH 12);<br>−23,2 mv (pH 2)	-	> 95%	ZetaSizer NANO, TEM
[39]	TiO_2 NPs	-	P: 21nm;<br>CM: 28.6 nm	−12.2 ± 0.25 mV	TiO_2: 0,563	LPS: NPs < 50 pg/mL; Culture media < 5 pg/mL)	DLS
	ZnO NPs		P: 10 nm;<br>CM: 16.9 nm	11.4 ± 0.17 mV	ZnO, 0,410		

[40]	Fe_3O_4	-	100 nm	-	-	-	-
[41]	MIONs	Cubic	P: 43 nm Solution: 43 nm	-	-	LPS < 0.25 EU ml^{-1})	TEM, X-ray diffraction, BET
[42]	CaP	Spherical or oval	1.84 ± 0,48 µm	−2,49 mV	-	-	SEM, EDS, DLS, XRD, ICP-OES
[43]	AuNPs	Spherical	5, 20, 80 nm	AuNPs-5: −22.01 ± 1.81 mV, AuNPs-20: −32.17± 2.19, AuNPs-80: −55.21± 7.34mV	-	-	TEM, UV-vis spectroscopy, DLS, Hyperspectral microscopy
[44]	PtNPs	Spherical, trian-gular, oval, and rod-shaped	40–50 nm	-	-	-	UV-vis spectroscopy, XRD, FITR, DLS, TEM, SEM
[45]	PdNPs	Spherical	~20 nm	-	-	-	UV-vis spectroscopy, XRD, FITR, DLS, TEM, SEM

[46]	Fe_3O_4 NPs	Spherical	8, 15, 30 nm	-	-	8 nm (99.9%), 15-20 nm (99.5%) and 20-30 nm (99.0%)	-
[47]	POSS NPs	Spherical	3–5 nm	-	-	-	FT-IR, TEM, NMR spectroscopy
[48]	nHAp	Rod-like	<100 nm	-	-	97%	SEM, EDS, X-ray diffraction, TEM
[49]	s-GO	-	50-500 nm	−55.9 ± 1.4 mV	-	LPS free	ZetaSizer NANO, TEM, AFM, micro-Rman spectrometer
[50]	PAMAM G2	-	G2: 3 nm	G2-NH_2: 19,8mV; G2-COOH: −21,7 mV; G2-OH: 4,8mV	-	-	DLS
	PAMAM G7		G7: 9nm	G7 NH_2: 30,1mV; G7-COOH:			

				−19,5mV; 2,8mV; G7-OH: 2,8mV			
[51]	NCs	-	Ag NCs: 1.3 nm; Fe_3O_4 NCs: 3.5 nm	-	-	-	TEM, DLS, UV-vis spectroscopy, EDS, FTIR

Table S6. Exposure conditions, and biological effects .

Reference	Experimental model[a]	Concentration	Time	Exposure conditions	Biological effects[e]
[20]	In vitro Human HMVECs	2–7 μg/mL	24 h	Immersion CM	PEI-SPION NPs uptake did not impact cell viability
[22]	In vivo Mouse BALB/c	20 μg in 50 μL PBS	3 times at daily inter-vals (day 0, 2, and 4)	Respiratory exposure (Intratracheal instillation)	NPs transferred across the pulmonary cell membrane and located in lysosomes

[24]	In Vitro Human THP-1	100 µg/mL	24 h	Immersion CM	-
	Clinical: Pneumoconiosis patients	-	-	Exposed to occupational inhaling	-
[25]	In vitro Human PBMCs	0.1, 1, 10 and 50 µM	24h	Immersion CM	Internalization of AuNPs in the early endosomes and/or in structures resembling MVB.
[26]	In vitro Mouse PMQ	10 µg/mL	0–48h	Immersion CM	↑ SWCNTs uptake with prolonged exposure time<br>No significant cell death<br>Alteration in primary macrophages morphology
[39]	In vitro Human PBMC	0.5–100 µg/mL	24 h	Immersion CM	TiO_2 NPs: No cell death<br>ZnO: No differences in inactivation and expression CD69 in T-cell.<br>↓ CD16 on NK-cells
	In vitro				

	Human MDDC				TiO_2 NPs: NPs active uptake. No alteration in surface markers<br>ZnO NPs: ↑ Cell death, ↑ Cas dose-dependent, ↑ DNA fragmentation. No NPs uptake and no change in surface marker expression
[40]	In Vitro Human BMSCs	400, 200, 100, 50, 25 µg/mL	1, 3, and 5 days	Immersion CM	↑ Cell viability in the optimal working concentration (50 µg/mL)
[41]	In vivo Mouse BALB/c	20 µg in 50 µL PBS	days 15, 17, and 19	Respiratory exposure (Intratracheal instillation)	NPs transferred across the pulmonary cell membrane and located in lysosomes<br>↑ Th1 polarization<br>↑ Tc1
	In vivo Mouse BALB/c OVA-sensitized				Mouse BALB/c:<br>↑ Activated Th + Tc<br>↑ Th1, ↑ Tc1<br>↑ IFN-γ, ↑ IL-4<br>↑ Inflammation
[42]	In vitro Mouse RAW264.7	250, 500, 1000, 2000 µg/mL	1–days	Immersion CM	No alteration in proliferation
	In vitro Human THP-1				No alteration in proliferation

[43]	In vitro Mouse mESCs	1 µg/mL	24–48 h	Immersion CM	LOEC: 5 µg/mL<br>Non-cytotoxic and does not induce ROS.<br>No interferences on self-renewal and pluripotency
[44]	In vitro Human A549	0, 2.5, 5, 10, 20, and 40 µmol/L	24h	Immersion CM	↑ Viability and proliferation<br>Morphological signals of autophagy<br>↑ ROS, ↑ Cas3, ↑ LDH, ↑ AchE
[45]	In vitro Human THP-1	5–25 µM	24 h	Immersion CM	-
[46]	In vitro Human iNPCs Cortical spheroids culture	0, 1, 10 and 100 µM	48 h	Immersion CM	Changes in morphology<br>No effects on cell viability, metabolic activity, neurodegeneration, and oxidative stress.
[47]	In vitro Human HUVECs:	0–600 ppm	24 and 48h	Immersion CM	↑ Viability, ↑ Migration, ↑ Wound healing, ↑ VEGFR-2, HSP-70, Ang-1, and Ang-2, ↑ miRNA-21 and miRNA-155, ↑ VEGF-A and TGF-β, ↓ miRNA-182
[48]	In vitro Mice C57BL/6 VSMCs	100µg/mL	24h, 7, and 14 days	Immersion CM	↑ ALP, Runx2, and OPN, ↑ Autophagic organelles, ↓ Lysosomal acidification, No effects on the viability, ↑ calcium deposition

[49]	In vitro<br>Rats Wistar<br>Astrocytes	10 µg / mL	6 days	Immersion<br>CM	No impair astrocyte morphology and cell density<br>No effect on viability<br>Did not cytotoxic effect
[50]	In vitro<br>Human<br>HUVECs	1–100 µg/mL	24h	Immersion<br>CM	G2: Low cytotoxicity<br>Moderate g1 arrest of cell cycle<br>G2-NH_2: ↑ ICAM-1(CD54), ↑ Apoptosis<br><br>G7: G7-NH_2: ↑ ICAM-1(CD54), ↑ PS<br>↑ Apoptosis<br>↑ Necrosis<br>↑ Plasma membrane blebbing, disintegration, and permeability<br>Moderate g1 arrest of cell cycle
[51]	In vitro<br>Human<br>L02<br><br>In vitro Human<br>HepG2	10–100<br>µmol/L	24h	Immersion<br>CM	Induce dose-dependent cytotoxicity<br>↓ Viability<br><br>Not difference in cytotoxicity

Table S7. Characterization of Extracellular Vesicles.

Reference	Biological origin and fluid collection	Isolation	EVs Nomenclature	Size	Characterization	EVs Enriched markers	Not enriched markers

[20]	HMVECs Cell culture conditioned media	HSC, MS	Extracellular Vesicles	100 ± 1000 nm	TEM, LSCM	-	-
[22]	Mouse BALF	Centrifugation and ultracentrifugation	Exosomes	30–90 nm	TEM, Protein dosage, FC	-	-
[24]	IMR-90 and THP-1 Cell culture conditioned media and Venous blood from patients (pneumoconiosis and control)	Ultracentrifugation	Exosomes	30–150 nm	TEM, NTA, miRNA Isolation and High-Throughput Sequencing, Immunoblotting RT-qPCR, Immunohistochemistry, FC	-	-
[25]	PBMCs Cell culture conditioned media	Centrifugation and ultracentrifugation	Exosomes	127.0 ± 3.8 nm	TEM, WB, Bradford protein assay, FC, SP-ICP-MS, NTA	TSG101, CD9, and CD81	-

[26]	PMQ Cell culture conditioned media	-	Exosomes<br>Extracellular Vesicles	50-100 nm<br>100-400 nm	TEM, SEM, RAMAN	-	-
[39]	PBMC and MDDC Cell culture conditioned media	Ultracentrifugation	Exosomes	30–100 nm	TEM, Protein concentration, NTA, FC	CD81, CD63, CD61, CD86, CD95/FasL MHCI and MHCII	
[40]	BMSCs Cell culture conditioned media	Centrifugation and ultracentrifugation	Exosomes	116.2 nm	TEM, NTA, WB	CD9, CD63, CD81, TSG101	Calnexin
[41]	Mouse BALF	Centrifugation and ultracentrifugation	Exosomes	30–90 nm	TEM, Protein dosage, WB, EDS, ICP-MS, FACS	TSG101	-

[42]	RAW264.7 and THP-1 Cell culture conditioned media	Total Exosomes Isolation Kit	Exosomes	30.2 ± 8.6 nm<br><br>Exossome aggregation: 196.3 ± 73.2	EXOCET kit, WB, ICP-OES, DLS	CD9, LAMP-1	-
[43]	mESCs Cell culture conditioned media	Ultracentrifugation and filtration	Extracellular vesicles	60–70 nm	TEM, WB, NTA, QCM-D, LC-MS/MS	CD63, HSP70, and Flotilina-1	Calreticulin
[44]	A549 Cell culture conditioned media	Differential centrifuga-tion and ExoQuick	Exosomes	90–100 nm	DLS, NTA, TEM, SEM, EXOCET™, FP, qRT-PCR, ELISA, BCA	TSG101, CD81, CD63, CD9	-
[45]	THP1 Cell culture conditioned media	Differential ultracentrifu-gation and ExoQuick™	Exosomes	50–80 nm.	DLS, NTA, SEM, TEM, EXOCET™, FP, BCA, qRT-PCR, Enzyme-linked immunosorbent assay, ELISA and WB	TSG101, CD9, CD63 and CD81	

[46]	iNPs Cortical spheroids Culture conditioned media	Differential ultracentrifu-gation and PEG-based method	Extracellular Vesicles	200–250 nm	RT-PCR, NTA, TEM	CD63, CD81, Alix, TSG101, Syntenin 1, ADAM10, RAB27b, and Syndecan	-
[47]	HUVECs Cell culture conditioned media	Exo-spin™ kit and centrifugation	Exosomes	-	SEM, TEM, FC, AChE activity	CD63	-
[48]	VSMCs Cell culture conditioned media	Centrifugation and ultracentrifugation	Exo-somes	100–133 nm	TEM, DLS, WB, granular analysis	Alix, TSG101, and CD9	GAPDH
[49]	Astrocytes Cell culture conditioned media	Centrifuga-tion	Microvesicles	50–500 nm	WB, LSCM, AFM, NTA, FTIR-ATR spectroscopy, UVRR	Flotillin-1	-
[50]	HUVECs Cell culture conditioned media	Centrifugation	Extracellular Vesicles	120 nm	NTA, TEM, FC	-	-

[51]	HepG2 Cell culture conditioned media	Centrifugation, filtration, and ultracentrifugation	Exosomes	50 nm	TEM, DLS, LSCM, EDS, SEM	-	-

Table S8. Modulation of extracellular vesicles and biological outcomes induced by nanomaterials.

Reference	Morphology	Size	Biogenesis	Cargo	Functional tests	Biological outcomes
[20]	-	-	↑ Extracellular vesicles biogenesis	-	NMCF7, 4T1 and HMVEC co-cultivated with EVs containing NMs: Intercellular transfer of NMs through EVs PS+ in	↑ EVs associated with apoptotic cell; Intercellular transfer of NMs through EVs PS+ in NMCF7, 4T1 or HMVEC co-cultivated with EVs
[22]	No alterations	No alterations	↑ Exosome biogenesis	-	**In vitro:** AM φ, Raw264.7, and iDCs cells co-cultivated with Exosomes: Exosomes are internalized<br><br>iDCs co-cultivated with Exosomes: ↑ iDC maturation and secretion of	↑ Exos biogenesis Exos MHCI H-2Kd+, MHCII I-Ad+, and CD80+ secreted are APCs origin.<br><br>**In vitro:** Exos are internalized by AM φ, Raw264.7, and iDCs cells co-cultivated with Exos. ↑ iDC maturation and secretion of cytokines DC1 and IL-12 Exos mediated.

					cytokines DC1 and IL-12<br><br>T-cells co-cultivated with Exosomes: ↑ Polarizing on T-cell differentiation in Th1 and Tc1 Exos mediated<br><br>AM φ co-cultivated with Exosomes: ↑ Phagocytic index of AM φ and secretion IL-12, and TNF- α<br><br>**In vivo:** Exosomes were distributed in the liver and spleen of BALB/c mice	↑ Polarizing on T-cell differentiation in Th1 and Tc1 Exos mediated.<br>↑ Phagocytic index of AM φ and secretion IL-12, and TNF- α Exos mediated.<br><br>**In vivo:** Exos were distributed in the liver and spleen of BALB/c mice.
[24]	-	-	↑ Exosome biogenesis	↓ hsa-let-7a-5p, ↓ hsa-let-7i-5p ↑ WASL expression	IMR-90 fibroblasts co-cultivated with Exosomes:<br>↑ Fibroblast trans-differentiation<br>↑ Collagen deposition<br>↑ Phagocytosis of NPs	↑ Exosomes biogenesis;<br>↓ hsa-let-7a-5p, ↓ hsa-let-7i-5p<br>↑ WASL expression; ↑ Phagocytosis of NPs<br>↑ Fibroblast transdifferentiation in IMR-90 fibroblasts co-cultivated with Exosomes

						↑ Collagen deposition in IMR-90 fibroblasts co-cultivated with Exosomes
[25]	-	↑ Exosomes size	No alterations	-	-	↑ Exosomes size and refractive index;<br>AuNPs internalized in Exosomes<br>PBMCs derived Exosomes eliminate AuNPs
[26]	-	-	↑ Exosome biogenesis	-	-	↑ Exosome biogenesis on the surface of macrophages;<br>Exocytosis of SWCNTs through Exosome and Extracellular vesicles;<br>Internalization sustained of SWCNTs in PMQ following exocytosis by Exosome
[39]	No alterations	No alterations	No alterations	No alterations	-	No alterations in Exosomes secretion, morphology, size, number, protein cargo
[40]	No alterations	No alterations	↑ Exosome biogenesis	MiR-21-5p (miRNA) foi regulado	**In vitro:**	↑ Exosomes biogenesis.

				positivamente em mag-BMSC-Exo	HUVECs and HSFs co-cultivated with Exosomes:<br>↑ Proliferation, migration, and angiogenesis<br>↑ miR-21-5p, ↓ SPRY2,<br>↑ PI3K/AKT and ERK1/2<br>↑ VEGF, HIF-1α, PDGFRα, and bFGF<br><br>**In vivo:**<br>Exosomes transplanted into skin wounds of mouses:<br>↑ Wound closure, ↑ Density of blood vessels, ↑ Collagen deposition, ↓ Scar widths,<br>↑ Angiogenesis, ↑ Formation of sebaceous glands and hair follicles	No morphological, size, shape, and electron density alterations<br><br>**In vitro:** ↑ Proliferation, migration, and angiogenesis in HUVECs and HSFs co-cultivated with Exosomes<br>↑ miR-21-5p, ↓ SPRY2,<br>↑ PI3K/AKT and ERK1/2<br>↑ Migration, proliferation, and tube formation in HUVECs co-cultivated with Exosomes<br>↑ Migration in HSFs co-cultivated with Exosomes<br>↑ Mature miR-21-5p, VEGF, HIF-1α, PDGFRα, and bFGF in HUVECs and HSFs co-cultivated with Exosomes<br><br>**In vivo**: ↑ Wound closure, ↑ Density of blood vessels, ↑ Collagen deposition, ↓ Scar widths,<br>↑ Angiogenesis, ↑ Formation of sebaceous glands and hair Follicles Exosomes induce
[41]	No alterations	No alterations	↑ Exosome biogenesis	-	**In vitro:**<br>Phagocytic and immune system cells co-cultivated with Exosomes:	No morphological and size changes on Exos<br><br>**In vitro:** ↑ Exos biogenesis. Exos induce iDCs maturation.

					Exosomes induce iDCs maturation and sensitized T-cell activation and differentiation	Exos induce sensitized T-cell activation and differentiation<br><br>**In vivo:** ↑ Exos biogenesis in the alveolar region of BALB/c mice.<br>Exos MHCI H-2Kd+, MHCII I-Ad+, CD80+, and CD86- secreted are APCs origin.<br>Exos induce systemic immune response through being eliminated from alveolar spaces.
[42]	-	-	↑ Exosome biogenesis	No altera-tions	-	↑ Exos biogenesis.<br>No alterations in Ca cargo;
[43]	↑ The rigid-ity of EVs	-	-	Differentiall y-expressed protein profile	**In vitro:**<br>4T1 co-cultivated with Exosomes:<br>↓ Proliferation and migration<br>↓ Cofilin expression and Erk phosphorylation	**EVs-5:** ↑ The rigidity of EVs, differentially-expressed protein profile, and cellular uptake.<br>↓ Proliferation and migration of 4T1 cells co-cultivated with Exos.<br>↓ Cofilin expression and Erk phosphorylation<br><br>**sEV-20 and sEV-80:** negligible effects.

[44]	No alterations	No alterations	↑ Exosomes biogenesis	↑ Exosomes total protein concentration ↑ Concentration of TSG101, CD9, CD63, and CD81 proteins,	-	↑ Exosomes biogenesis ↑ Exosomes total protein concentration ↑ Concentrations of TSG101, CD9, CD63, and CD81 proteins, Typical morphology and no significant difference in size was observed.
[45]	No alterations	-	↑ Exosomes biogenesis	↑ Exosomes cytokine and chemokine levels (IL-6, MCP-1, IL-8, GM-CSF, TNF-α and IL-1β)	-	↑ Exos biogenesis. ↑ Exos cytokine and chemokine levels (IL-6, MCP-1, IL-8, GM-CSF, TNF-α and IL-1β). ↑ TSG101, CD9, CD63, and CD81 Exos markers expression levels; No morphological changes were observed.
[46]	No alterations	↓ Extracellular Vesicles mean size	↑ Exosomes biogenesis	-	-	**8 and 15 nm:** ↑ EVs biogenesis. **30 nm:** ↓ EVs mean size<br><br>No morphological changes were observed. Differential gene expression of EV biogenesis markers (CD63,

						CD81, Alix, TSG101, Syntenin1, ADAM10, RAB27b, and Syndecan) by different size NPs.
[47]	-	-	↑ Exosomes biogenesis	-	-	↑ Exosomes biogenesis
[48]	-	-	↑ Exosomes biogenesis	-	-	↑ Exosomes biogenesis<br>↑ Ca cargo
[49]	No alterations	No alterations	↑ Microvesicles biogenesis	Altered protein content in EVs	**In vitro:**<br>Neurons co-cultivated with MVs:<br>↑ PSCs, ↓ Neuronal stiffness<br>↑ Synaptic activity	↑ Microvesicles biogenesis;<br>Altered protein cargo in Microvesicles;<br>No alteration in Microvesicles morphology and size;<br>↑ PSCs in cortical neurons co-cultivated with Microvesicles<br>↓ Neuronal stiffness.<br>↑ Synaptic activity
[50]	-	-	↑ Extracellular Vesicles biogenesis	-	-	↑ EVs biogenesis;<br>↑ EVs CD105+, PS+, TOM20+
[51]	No alterations morphology	No alterations	-	-	**In vitro:**	No changes in Exosomes morphology and size

Change in surface charge		HepG2 and U87 co-cultivated with Exosomes:<br>↓ Viability,<br>↑ ROS,<br>↓ Cellular uptake	Change in Exosomes surface charge<br><br>↓ Viability in HepG2 and U87 co-cultivated with Exosomes<br><br>Cellular uptake of Exosomes HepG2 and U87<br>↑ ROS in HepG2 co-cultivated with Exosomes

Table S9. Report of methodological parameters and EV-TRACK record.

Reference	Reported Parameters	Parameters not reported	EV-METRIC
[20]	Electron microscopy images; Specific ultracentrifuge.	Density gradient; EV density; EV-enriched proteins and non EV-enriched protein; Specific ultracentrifuge; Qualitative and quantitative analysis.	Not registered
[22]	Specific ultracentrifuge;EV-enriched proteins ;<br>Electron microscopy images;<br>Qualitative analysis.	Density gradient; EV density; non EV-enriched protein-; Quantitative analysis.	Not registered

[24]	Specific ultracentrifuge; Qualitative and quantitative analysis; Electron microscopy images .	Density gradient; EV density; EV-enriched proteins andnon EV-enriched protein .	Not registered
[25]	Specific ultracentrifuge; Lysate preparation; Qualitative and quantitative analysis; EV-enriched proteins-.	Density gradient; EV density;Electron microscopy images-; non EV-enriched protein.	Not registered
[26]	Not applicable.	Density gradient; EV density; EV-enriched proteins; Specific ultracentrifuge; Qualitative and quantitative analysis; Electron microscopy images.	Not registered
[39]	EV-enriched proteins; Electron microscopy images; Specific ultracentrifuge; Qualitative and quantitative analysis.	Density gradient; EV density.	57%
[40]	Specific ultracentrifuge; Qualitative and quantitative analysis;Electron microscopy images; EV-enriched protein andnon EV-enriched protein ; Lysate preparation.	Density gradient.	Not registered

[41]	Not applicable.	Density Gradient; EV density; Specific ultracentrifuge; EV-enriched proteins andnon EV-enriched protein; Qualitative and quantitative analysis;Electron microscopy images.	0%
[42]	Specific ultracentrifuge; Qualitative and quantitative analysis; EV-enriched proteins; Lysate preparation.	Qualitative analysis; Density gradient; EV density;Electron microscopy images .	Not registered
[43]	EV-enriched proteins and non EV-enriched protein; Electron microscopy images ; EV density; Qualitative and quantitative analysis.	Density Gradient; Specific ultracentrifuge; Lysate preparation.	Not registered
[44]	EV density;Electron microscopy images; Qualitative and quantitative analysis; EV-enriched proteins .	Density Gradient; Specific ultracentrifuge; non EV-enriched protein; Lysate preparation.	Not registered
[45]	EV Density; Electron microscopy images; Qualitative and quantitative analysis; EV-enriched proteins .	Density Gradient; Specific ultracentrifuge; ; non EV-enriched protein; Lysate preparation.	Not registered

[46]	EV Density; Electron microscopy images; Qualitative and quantitative analysis<br>EV-enriched proteins; Specific ultracentrifuge.	Density Gradient; non EV-enriched protein; Lysate preparation.	Not registered
[47]	Electron microscopy images-; Specific ultracentrifuge; Qualitative and quantitative analysis;_EV-enriched proteins-.	EV density; Density gradient;Non EV-enriched protein; Lysate preparation.	Not registered
[48]	Specific ultracentrifuge;_Electron microscopy images; Qualitative and quantitative analysis; EV-enriched proteins and non EV-enriched proteins; EV density.	Density gradient; Lysate preparation.	Not registered
[49]	Specific ultracentrifuge; Qualitative and quantitative analysis.	EV-enriched proteins andnon EV-enriched protein ;Electron microscopy images; EV; Lysate preparation.	Not registered
[50]	Specific ultracentrifuge; EV density; Quantitative analysis.	Density gradient; EV density; Qualitative analysis;Electron microscopy images; EV-enriched protein and non EV-enriched protein.	Not registered

[51]	Electron microscopy images; Specific ultracentrifuge; Qualitative and quantitative analysis.	Density gradient; EV density; EV-enriched proteins andnon EV-enriched protein.	Not registered

Table S10. Overall score and categorization of the reliability of studies (ToxRTool).

Reference	Score	Category A	Category B
[20]	17	2	3
[22]	10	3	3
[24]	10	3	3
[25]	13	2	3
[26]	12	2	3
[39]	18	1	1
[40]	16	1	1
[41]	15	2	3
[42]	14	2	2
[43]	17	1	1
[44]	16	1	3
[45]	17	1	3
[46]	15	1	3

[47]	18	1	3
[48]	17	1	3
[49]	16	1	1
[50]	16	1	1
[51]	13	2	3

Table S11. Analysis of compliance in reporting animal research (ARRIVE 2.0 checklist).

Description of items	[20]			[40]			[41]			[47]		
	Yes	No	Partially	Yes	No	Partially	Sim	No	Partially	Sim	No	Partially
1. Study Design	X			X			X					X
2. Sample Size			X			X			X			X
3. Inclusion & Exclusion Criteria		X		X				X			X	
4. Randomisation	X			X			X				X	
5. Blinding	X				X		X				X	

6. Outcomes Measures	X		X	X	X	
7. Statistical Methods	X		X	X	X	
8. Experimental Animals	X		X	X		X
9. Experimental Procedures		X	X	X		
10. Results	X		X	X	X	

Table S12. Quality assessment of case-control studies (Newcastle-Ottawa Scale (NOS)).

Reference	Selection	Comparability	Exposure	Score
[22]	★★★[1]	★★	★★★	8/9

[1] Newcastle-Ottawa Scale (NOS) "Star system" in which a study is judged from three broad perspectives: the selection of study groups; the comparability of the groups; and verification of exposure or outcome of interest for case-control or cohort studies, respectively. For each attribute met, the study receives one star, with a maximum number of stars: selection (0–4 points), comparability (0– 2 points) and exposure (0– 3 points).
